# Lightweight Pre-Trained Korean Language Model Based on Knowledge Distillation and Low-Rank Factorization

**DOI:** 10.3390/e27040379

**Published:** 2025-04-02

**Authors:** Jin-Hwan Kim, Young-Seok Choi

**Affiliations:** 1 Korea Telecom Corporation Agentic AI Lab, Seongnam-si 13606, Republic of Korea; 2 Department of Electronics and Communications Engineering, Kwangwoon University, Seoul 01897, Republic of Korea

**Keywords:** natural language processing, pre-trained language model, Korean language model, knowledge distillation, low-rank factorization, resource-constrained environment

## Abstract

Natural Language Processing (NLP) stands as a forefront of artificial intelligence research, empowering computational systems to comprehend and process human language as used in everyday contexts. Language models (LMs) underpin this field, striving to capture the intricacies of linguistic structure and semantics by assigning probabilities to sequences of words. The trend towards large language models (LLMs) has shown significant performance improvements with increasing model size. However, the deployment of LLMs on resource-limited devices such as mobile and edge devices remains a challenge. This issue is particularly pronounced in languages other than English, including Korean, where pre-trained models are relatively scarce. Addressing this gap, we introduce a novel lightweight pre-trained Korean language model that leverages knowledge distillation and low-rank factorization techniques. Our approach distills knowledge from a 432 MB (approximately 110 M parameters) teacher model into student models of substantially reduced sizes (e.g., 53 MB ≈ 14 M parameters, 35 MB ≈ 13 M parameters, 30 MB ≈ 11 M parameters, and 18 MB ≈ 4 M parameters). The smaller student models further employ low-rank factorization to minimize the parameter count within the Transformer’s feed-forward network (FFN) and embedding layer. We evaluate the efficacy of our lightweight model across six established Korean NLP tasks. Notably, our most compact model, KR-ELECTRA-Small-KD, attains over 97.387% of the teacher model’s performance despite an 8.15× reduction in size. Remarkably, on the NSMC sentiment classification benchmark, KR-ELECTRA-Small-KD surpasses the teacher model with an accuracy of 89.720%. These findings underscore the potential of our model as an efficient solution for NLP applications in resource-constrained settings.

## 1. Introduction

Natural Language Processing (NLP) has rapidly evolved, aiming to enable machines to process human language [1]. This has led to significant advancements in human–computer interaction. Language models (LMs) are core to NLP, capturing language structure and meaning by learning the probability distribution of word sequences [2]. Since around 2018, pre-trained language models (PLMs) [3] have reshaped the NLP landscape. Trained on vast corpora of unlabeled text, they learn rich, general-purpose language representations. These models are trained on vast corpora of unlabeled text, enabling them to learn rich, general-purpose representations of language that can be fine-tuned for specific downstream tasks. A key innovation that has propelled the development of PLMs is the Transformer architecture, introduced by Vaswani et al. [4]. The Transformer’s self-attention mechanism allows for parallel processing of input sequences, resulting in substantial improvements in training efficiency and model performance. This architecture serves as the foundation for influential models like BERT [3], which leverages the Transformer’s encoder to generate contextualized word embeddings, and GPT [5], which utilizes the Transformer’s decoder for language generation.

A notable trend in NLP research has been the continuous growth in the size of language models, leading to the development of large language models (LLMs) [6,7,8,9,10,11]. This increase in scale has generally been accompanied by improvements in performance across a variety of NLP tasks. However, the substantial computational resources required by LLMs present a major obstacle to their widespread deployment, especially in resource-constrained environments such as mobile devices and edge computing platforms. The computational demands of LLMs often surpass the capabilities of these devices, hindering their practical use in such contexts.

Despite the remarkable progress made with LLMs, the development of pre-trained models for languages other than English has lagged behind. This disparity is particularly evident for the Korean language, where the availability of such models remains scarce. The limited development of pre-trained Korean language models hinders the advancement of NLP research and applications tailored to the unique characteristics of the Korean language. Korean poses specific challenges for language modeling due to its agglutinative morphology, where words are formed by combining multiple morphemes, and its relatively free word order compared to English.

Recognizing these challenges, researchers have explored various methods for creating lightweight PLMs that are more suitable for resource-constrained environments. These methods include pruning, quantization, and knowledge distillation [12,13,14,15]. Pruning involves removing less important connections in a neural network, thereby reducing the number of parameters and computational operations [16]. Quantization reduces the precision used to represent model parameters, for example, from 32-bit floating-point to 8-bit or 4-bit integers, leading to a smaller model size and faster inference [17]. Knowledge distillation, on the other hand, trains a smaller “student” model to mimic the behavior of a larger, more accurate “teacher” model [12,14]. This allows the student model to achieve performance comparable to the teacher model while having a smaller size and faster inference speed. Other notable compression techniques include DistilBERT [18], TinyBERT [19], and ALBERT [20], each employing different strategies for model size reduction. Sanh et al. introduced DistilBERT, which applied knowledge distillation to the BERT model, achieving 96% of BERT’s performance with 40% fewer parameters [18].

Building upon these advancements, we propose a novel lightweight pre-trained Korean language model based on knowledge distillation and low-rank factorization and demonstrating a novel, effective combination of these techniques for the under-resourced Korean language. Our model is specifically designed to address the scarcity of pre-trained models for Korean while also being efficient enough for deployment on resource-limited devices. In our approach, we first train a large “teacher” model, a Korean ELECTRA model with 432 MB (approximately 110 M parameters), which serves as a knowledge source. We then employ knowledge distillation to transfer the knowledge from this teacher model to a smaller “student” model, with sizes of 53 MB (≈14 M parameters), 35 MB (≈13 M parameters), 30 MB (≈11 M parameters), and 18 MB (≈4 M parameters). To further reduce the model size, we apply low-rank factorization to the student models with sizes of 35, 30, and 18 MB, which reduces the number of parameters in the feed-forward network (FFN) and embedding layer of the Transformer.

Our empirical studies involve a comprehensive investigation of various knowledge distillation and low-rank factorization strategies. We evaluate the performance of our lightweight Korean ELECTRA model, which we call KR-ELECTRA-Small-KD, on six different Korean NLP tasks, which are widely used for evaluating Korean language models. Notably, our KR-ELECTRA-Small-KD model, despite being 8.15 times smaller than the teacher model (KR-ELECTRA-Base), achieves more than 97.387% of the teacher model’s performance. In particular, on the NSMC single sentence binary classification task, KR-ELECTRA-Small-KD achieves a test accuracy score of 89.720, which is even higher than that of the teacher model.

The remainder of this paper is structured as follows: Section 2 details the methodology behind our proposed lightweight Korean language model. Section 3 describes the six Korean NLP tasks used for evaluation and presents the experimental results. Finally, Section 4 concludes the paper, highlighting the contributions and implications of our work.

## 2. Methods

This section details the methodology underpinning our lightweight pre-trained Korean language model. Our approach integrates two principal techniques: knowledge distillation and low-rank factorization. We first train a large “teacher” model based on the Korean ELECTRA architecture. Subsequently, we employ knowledge distillation to transfer the knowledge from this teacher model to a smaller “student” model. Finally, we apply low-rank factorization to specific components of the student model to further reduce its size while maintaining performance.

### 2.1. Teacher Model Training

The foundation of our approach is a large pre-trained Korean ELECTRA model, which serves as the “teacher” model [18]. We choose ELECTRA over BERT due to its superior pre-training efficiency. The teacher model is trained on an extensive corpus of Korean text, encompassing diverse genres such as news articles, web documents, and online conversations. Standard preprocessing techniques, including tokenization, normalization, and segmentation, are applied to the training data.

Our teacher model, designated as KR-ELECTRA-Base, has a size of 432 MB. It comprises 12 Transformer layers, each with a hidden size of 768 and 12 attention heads. The model is trained using the hyperparameters specified in the original ELECTRA [18]. The training objective is to minimize the masked language modeling (MLM) loss. During training, a portion of the input tokens is randomly masked, and the model is trained to predict the original tokens that were masked.

### 2.2. Knowledge Distillation

Following the teacher model training, we leverage knowledge distillation to transfer its learned knowledge to a smaller, more efficient “student” model [12]. The core principle of knowledge distillation is to train the student model to emulate the output probabilities of the teacher model, along with matching the ground-truth labels. This enables the student model to learn not only the correct answers but also the subtle nuances captured in the teacher’s predictions, including the relative probabilities assigned to incorrect answers.

**Figure 1:**
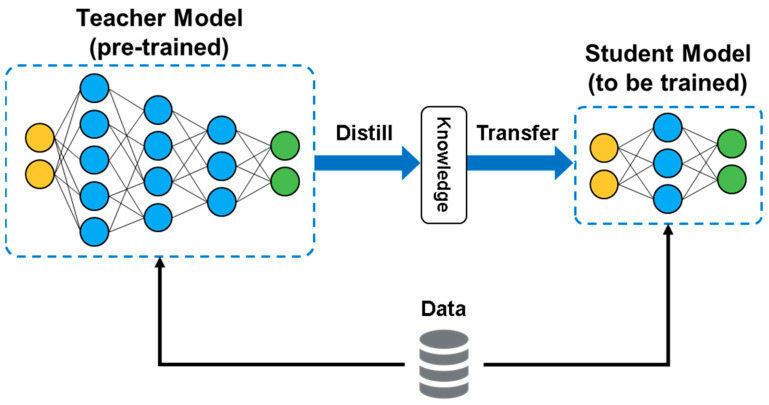
Illustration of the knowledge distillation process employed to transfer knowledge from the large teacher model to the smaller student model.

We employ four distinct types of knowledge distillation losses, as depicted in Figure 1:

First, for an embedding-layer distillation, the loss function encourages the student model to produce embedding representations that are similar to those generated by the teacher model. It is quantified as the mean squared error (MSE) between the student’s and teacher’s embedding matrices, after applying a linear transformation to align their dimensions. This approach, aligning embedding vectors using MSE loss, follows prior work on layer-wise representation alignment in Transformer compression studies [14,21]:

(1) $$L_{embedding}=MSE(E^{S}W_{e},E^{T})$$

where the matrices $E^{S}\in R^{l\times d_{e}^{S}}$ and $E^{T}\in R^{l\times d_{e}^{T}}$ refer to the embedding matrix of student and teacher models, respectively, and $W_{e}\in R^{d_{e}^{S}\times d_{e}^{T}}$ is a learnable linear Transformation matrix, which transforms the embedding matrix of student model into the same space as the embedding matrix of teacher model, and MSE(·) denotes the mean squared error loss function. The scalar value $d_{e}^{S}$ and $d_{e}^{T}$ denote the embedding sizes of student and teacher models, and l is the number of input text length.

Second, for a hidden states based distillation, the loss function aims to align the hidden representations of the student model with those of the teacher model across all Transformer layers. It is computed as the average MSE between the student’s and teacher’s hidden states across all layers. The loss is expressed as follows:

(2) $$L_{hidden}=\frac{1}{tl-1}\sum_{i=1}^{tl-1}MSE(H_{i}^{S}W_{h},H_{i}^{T})$$

where the matrices $H_{i}^{S}\in R^{l\times d_{h}^{S}}$ and $H_{i}^{T}\in R^{l\times d_{h}^{T}}$ refer to the hidden states corresponding to the i-th Transformer layer of student and teacher model, $W_{h}\in R^{d_{h}^{S}\times d_{h}^{T}}$ is a learnable linear transformation playing a similar role as $W_{e}$, and tl is the number of layers. The scalar value $d_{h}^{S}$ and $d_{h}^{T}$ denote the hidden sizes of student and teacher models. In this study, we used the average of the loss values for each Transformer layer.

**Figure 2:**
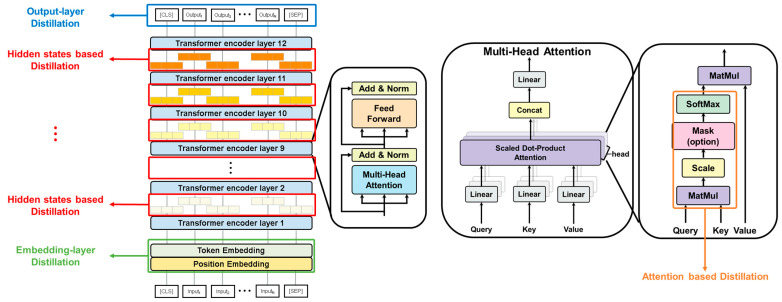
Detailed representation of the knowledge distillation process, illustrating the four targeted components: embedding layer, hidden states, output layer, and attention matrix.

In the field of Natural Language Processing, knowledge distillation has often been applied for model compression, particularly with BERT-based models [21,22]. However, this study employs knowledge distillation in the context of ELECTRA, a model known for its efficient pre-training. We utilize four distinct types of knowledge distillation losses, specifically targeting the embedding layer, hidden states, output layer, and attention mechanisms within the Transformer encoder, as illustrated in Figure 2.

Third, the output-layer distillation utilizes the loss function which encourages the student model to generate output probabilities that closely resemble those produced by the teacher model. It is measured using the negative cosine similarity between the student’s and teacher’s output probabilities. The loss is defined as follows:

(3) $$L_{output}=1-\frac{O^{S}W_{o}\cdot O^{T}}{\left\|O^{S}W_{o}\right\|\left\|O^{T}\right\|}$$

where the matrices $O^{S}\in R^{l\times d_{h}^{S}}$ and $O^{T}\in R^{l\times d_{h}^{T}}$ refer to the last output of Transformer layer of student and teacher model, $W_{o}\in R^{d_{h}^{S}\times d_{h}^{T}}$ is a learnable linear Transformation playing a similar role as $W_{e}$ and $W_{h}$. The loss value always belongs to the interval [0,2] because two proportional vectors have a cosine similarity of 1 and two opposite vectors have a cosine similarity of −1.

Then, the attention-based distillation uses an attention matrix indicating the degree of relationship between each input token. This loss function focuses on aligning the attention patterns learned by the student model with those learned by the teacher model. Aligning attention maps using KL-divergence builds upon techniques used in other Transformer distillation frameworks [21,22], helping the student model capture contextual relationships. It is calculated as the average Kullback–Leibler (KL) divergence between the student’s and teacher’s attention matrices across all attention heads and layers. The loss is given by

(4) $$A=softmax\left(\frac{QK^{T}}{\sqrt{d_{k}}}\right)$$

where $Q\in R^{l\times d_{k}}$ and $K\in R^{l\times d_{k}}$ refer to query and key, $d_{k}$ is number of attention head size, and it is computed by a compatibility function of the query with the corresponding key. The attention based distillation uses Kullback–Leibler divergence (KL-divergence) to calculate the difference between the two probability distributions, and the objective is defined as follows:

(5) $$L_{attention}=\frac{1}{h}\sum_{j=1}^{h}D_{KL}(A_{j}^{S},A_{j}^{T})$$

where $A_{i}^{S}\in R^{l\times l}$ and $A_{i}^{T}\in R^{l\times l}$ refer to the attention matrix corresponding to the j-th attention head of student and teacher model, h is number of attention head, and $D_{KL}(\;)$ means KL-divergence function.

The total knowledge distillation loss is a weighted combination of these four individual losses which is given by

(6) $$L_{KD}={\alpha_{1}L}_{embedding}+{\alpha_{2}L}_{hidden}+{\alpha_{3}L}_{output}+{\alpha_{4}L}_{attention}$$

where $\alpha_{1}$, $\alpha_{2}$, $\alpha_{3},$ and $\alpha_{4}$ are hyperparameters that determine the relative importance of each loss term. In our experiments, these hyperparameters are set to ensure that each loss term contributes equally to the overall knowledge distillation loss.

### 2.3. Low-Rank Factorization

Two structures within the Transformer architecture that contain a large number of parameters are the feed-forward network (FFN) and the embedding layer. For example, In the ELECTRA model of the base model size, the number of parameters of FFN is 56,623,104, which is shown in Table 1.

To further reduce the model size, we apply low-rank factorization to the student models with sizes of 35, 30, and 18 MB [17]. Low-rank factorization decomposes a large matrix into a product of two smaller matrices, thus reducing the number of parameters. We apply this technique to the FFN and the embedding layer of the Transformer, as these components account for a significant portion of the model’s parameters.

**Figure 3:**
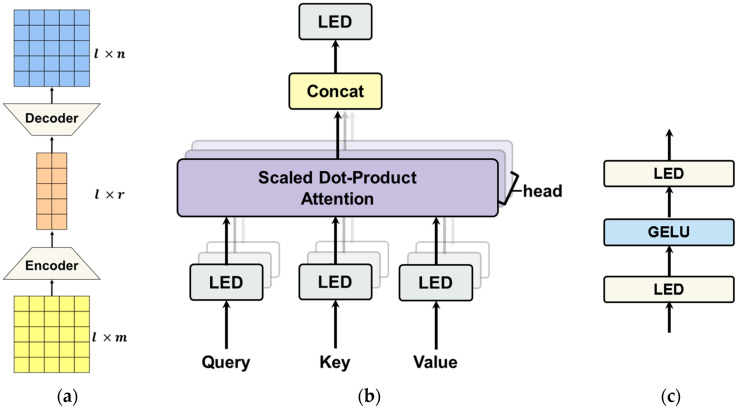
Detailed architecture of the lightweight Transformer components enhanced with low-rank factorization: (**a**) linear encoder–decoder (LED) within the feed-forward network (FFN), (**b**) lightweight multi-head attention (LMHA), and (**c**) the modified FFN.

Figure 3 shows the structure of light Transformer based on low-rank factorization. In Figure 3a, the linear encoder–decoder (LED) consists of two steps. (1) First step: the input of l×m dimension is converted into l×r dimension through the linear encoder; (2) second step: the input of l×r dimension is converted into l×n dimension through the linear decoder. When we apply the linear encoder–decoder to the Transformer, the value of r is essentially set to a value that satisfies the condition of r≪m,n. Figure 3b,c indicate lightweight multi-head attention (LMHA) and lightweight feed-forward network (LFFN) in which the number of parameters is reduced by changing the linear layer to a linear encoder–decoder.

If the value of r is set to $\frac{m}{4}$, the number of parameters of LFFN is 14,155,776, which is shown in Table 2. In our experiment, we find that $r=\frac{m}{4}$ performs well. The low-rank matrices were initialized using Singular Value Decomposition (SVD) of the corresponding weight matrices in the pre-trained teacher model.

Three variants of lightweight Korean language model are developed, designated as KR-ELECTRA-Small-LF-V1, KR-ELECTRA-Small-LF-V2, and KR-ELECTRA-Small-LF-V3, with model sizes of 35 MB, 30 MB, and 18 MB, respectively. These models differ in the extent of low-rank factorization applied:

- KR-ELECTRA-Small-LF-V1 (35 MB): In this variant, low-rank factorization is applied exclusively to the FFN.
- KR-ELECTRA-Small-LF-V2 (30 MB): Here, low-rank factorization is applied to both the FFN and the multi-head attention mechanism.
- KR-ELECTRA-Small-LF-V3 (18 MB): This variant extends the application of low-rank factorization to the FFN, the multi-head attention mechanism, and the embedding layer.

**Figure 4:**
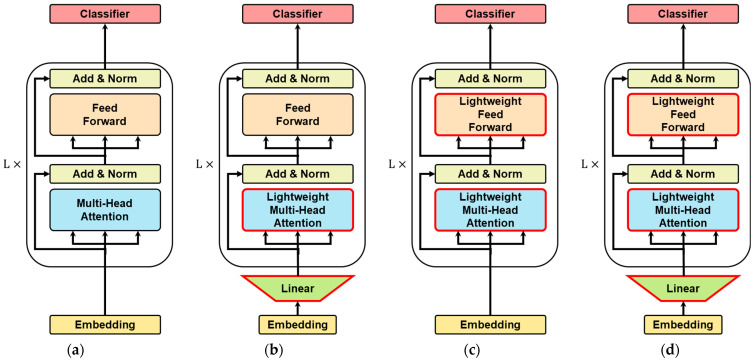
Illustration of the four model variants. (**a**) Original ELECTRA architecture. (**b**) Lightweight ELECTRA V1 with low-rank factorization applied to the FFN. (**c**) Lightweight ELECTRA V2 with low-rank factorization applied to the FFN and multi-head attention. (**d**) Lightweight ELECTRA V3 with low-rank factorization applied to the FFN, multi-head attention, and embedding layer.

Figure 4 shows illustration of four models. Figure 4a is an existing ELECTRA structure and Figure 4b–d are three versions of the lightweight ELECTRA, respectively.

## 3. Experiments and Discussion

This section presents the experimental setup, datasets, and results of our evaluation of the proposed lightweight pre-trained Korean language model. We conduct comprehensive experiments to assess the performance of our model on six diverse Korean NLP tasks. We also analyze the impact of different knowledge distillation and low-rank factorization strategies on model performance and size.

### 3.1. Experimental Setup

We trained our models using the Google Cloud Platform (GCP) and utilized cloud TPUs for accelerated computation, supported by the TensorFlow Research Cloud (TFRC) program. We used the same hyperparameters as the original ELECTRA model for training our teacher model (KR-ELECTRA-Base) [18]. The teacher model was fine-tuned separately for each downstream task using the hyperparameters detailed below. For the student models, we utilized the AdamW optimizer and experimented with different hyperparameter settings for knowledge distillation and low-rank factorization, as detailed in Section 2. The full set of pre-training hyperparameters are listed in Table 3. The detailed training configurations for each downstream task during fine-tuning are shown in Table 4.

### 3.2. Datasets

We evaluate our models on six widely used Korean NLP tasks, covering a range of language understanding abilities:

1. Naver Sentiment Movie Corpus (NSMC) [23]: A binary sentiment classification task, where the goal is to predict whether a given movie review is positive or negative. The dataset consists of 200,000 movie reviews collected from the Naver movie review website. Each review is shorter than 140 characters. The numbers of positive and negative reviews are balanced. Examples are shown in Table 3.
2. Korean Hate Speech Dataset (KOHATE) [24]: A binary classification task, where the goal is to identify hate speech in online comments. The dataset consists of 9381 comments from Korean entertainment news aggregation platforms, annotated for the existence of social bias and hate speech.
3. Korean Natural Language Inference (KorNLI) [25]: A natural language inference (NLI) task, where the goal is to determine the relationship between a premise and a hypothesis (entailment, contradiction, or neutral). The dataset consists of 570,000 sentence pairs, translated from the English MultiNLI dataset.
4. Korean Semantic Textual Similarity (KorSTS) [25]: A semantic textual similarity (STS) task, where the goal is to predict the similarity score between two sentences. The dataset consists of 8628 sentence pairs, translated from the English STS Benchmark dataset.
5. Named Entity Recognition (NER) [26]: We use a Korean NER dataset collected from NAVER [26]. The dataset consists of sentences annotated with 14 entity types. The goal is to identify and classify named entities in the text.
6. Korean Question Answering Dataset (KorQuAD) 1.0 [27]: A machine reading comprehension (MRC) task, where the goal is to answer a question given a passage of text. The dataset consists of over 60,000 question–answer pairs based on Korean Wikipedia articles. Each piece of data consists of a passage, a question, and a starting point and ending point for the correct answer. It is structured in the same way as the Stanford Question Answering Dataset (SQUAD v1.0).

Table 5 and Table 6 provide examples of the NSMD dataset and KorNLI dataset, respectively.

### 3.3. Results

We present the results of our experiments in Table 7, Table 8, Table 9 and Table 10. As shown in the “Avg” column of the tables, our most compact model, KR-ELECTRA-Small-LF-V3, achieves over 93.989% of the teacher model’s average performance, while KR-ELECTRA-Small-KD achieves over 97.387%. Table 7 shows the performance of our KR-ELECTRA-Small-KD model, which is trained using knowledge distillation without low-rank factorization. Table 8, Table 9 and Table 10 show the performance of our KR-ELECTRA-Small-LF models, which are trained using both knowledge distillation and low-rank factorization.

#### 3.3.1. Knowledge Distillation Results (KR-ELECTRA-Small-KD)

Table 7 shows that KR-ELECTRA-Small-KD achieves competitive performance compared to the teacher model (KR-ELECTRA-Base) across all six tasks, despite being 8.15 times smaller. Notably, on the NSMC task, KR-ELECTRA-Small-KD outperforms the teacher model, achieving an accuracy of 89.720% compared to 89.324% for the teacher model. This demonstrates the effectiveness of knowledge distillation in transferring knowledge from a large teacher model to a smaller student model.

We also observe that the performance of KR-ELECTRA-Small-KD varies depending on the hyperparameter β, which controls the balance between the knowledge distillation loss and the original ELECTRA pre-training loss. A value of β of 0.5 generally yields the best performance, suggesting that a balance between retaining the teacher’s knowledge and adapting to the smaller model size is crucial.

#### 3.3.2. Low-Rank Factorization Results (KR-ELECTRA-Small-LF)

Table 8, Table 9 and Table 10 demonstrate the impact of low-rank factorization on model performance and size. We observe that applying low-rank factorization to the FFN (KR-ELECTRA-Small-LF-V1) results in a slight decrease in performance compared to KR-ELECTRA-Small-KD, but significantly reduces the model size from 53 MB to 35 MB. Applying low-rank factorization to both the FFN and the multi-head attention mechanism (KR-ELECTRA-Small-LF-V2) further reduces the model size to 30 MB, but with a more noticeable drop in performance. Finally, applying low-rank factorization to the FFN, multi-head attention, and the embedding layer (KR-ELECTRA-Small-LF-V3) results in the smallest model size of 18 MB, but with the largest performance drop.

These results indicate a trade-off between model size and performance when applying low-rank factorization. While low-rank factorization can significantly reduce model size, it can also lead to a decrease in performance if applied too aggressively.

#### 3.3.3. Comparison with Other Models

We also compare our models with other existing models of similar sizes. We compare KR-ELECTRA-Small-KD (53 MB) with the original ELECTRA-Small model (53 MB) and KR-ELECTRA-Small-LF-V3 (18 MB) with KR-ELECTRA-Tiny (17 MB).

Table 7 shows that KR-ELECTRA-Small-KD outperforms ELECTRA-Small on all six tasks, demonstrating the effectiveness of knowledge distillation. Table 10 shows that KR-ELECTRA-Small-LF-V3 outperforms KR-ELECTRA-Tiny on most tasks, despite having a similar model size. This highlights the benefits of using both knowledge distillation and low-rank factorization for creating lightweight models.

To provide a more comprehensive context, we consider established compressed models like TinyBERT, DistilBERT, and ALBERT. These models also employ techniques like knowledge distillation and parameter reduction but differ in specific approaches (e.g., TinyBERT’s two-stage distillation, ALBERT’s parameter sharing). While a direct experimental comparison on Korean tasks is beyond this revision’s scope due to resource limitations, we compare qualitatively. DistilBERT [18] focuses on output probabilities, whereas our method uses a broader set of distillation targets. TinyBERT requires extensive two-stage training. ALBERT primarily uses parameter sharing. Our work demonstrates a specific, effective combination of KD and LF tailored for Korean. We acknowledge that direct comparisons would further strengthen our findings and plan this for future work.

#### 3.3.4. Inference Time

To demonstrate the practical efficiency gains of our proposed models, we measured their inference times. We report the average inference time per sentence (in milliseconds) for the teacher model and each student model on a CPU environment (Intel Xeon Platinum 8275CL @ 3.00 GHz), processing a single sentence at a time. The results are summarized in Table 11.

#### 3.3.5. Ablation Study on Low-Rank Factorization

To evaluate the impact of low-rank factorization, we conducted an ablation study by comparing models with and without this technique applied to the FFN. Table 12 presents the results of this analysis, where “Without LF” corresponds to the KR-ELECTRA-Small-KD model (53 MB), and “With LF” corresponds to the KR-ELECTRA-Small-LF-V1 model (35 MB, LF applied to FFN only).

As shown in Table 12, applying low-rank factorization to the FFN results in a slight performance decrease across tasks (average drop of approx. 2.47 points) but yields a substantial model size reduction (from 53 MB to 35 MB). This highlights the trade-off between model compression and performance. Further applying LF to attention and embeddings (LF-V2, LF-V3) continues this trend, further reducing size at the cost of accuracy, as seen in Table 8 and Table 9. The choice between these variants depends on the specific resource constraints and performance requirements of the target application.

#### 3.3.6. Impact of Distillation and Factorization Strategies

Our knowledge distillation approach leverages four distinct losses (embedding, hidden, output, attention). The strong performance of KR-ELECTRA-Small-KD (Table 7), even outperforming the teacher on NSMC, suggests that this multi-faceted distillation effectively transfers diverse aspects of the teacher’s knowledge. The subsequent application of low-rank factorization offers further compression but requires careful consideration of the trade-off, as discussed above. The choice of rank (r = m/4 in our case) is a critical hyperparameter that likely influences this balance.

### 3.4. Discussion

In this section, we provide a more thorough discussion of our proposed lightweight Korean ELECTRA model, focusing on both the architectural choices and the experimental results. First, the adoption of low-rank factorization (LF) in the feed-forward network (FFN), multi-head attention, and embedding layer significantly reduce the parameter count, as summarized in Table 2 and Table 10. This strategy effectively addresses resource constraints on mobile or edge devices while retaining acceptable performance levels across six benchmark tasks. However, we observe that extensive factorization in the embedding layer (KR-ELECTRA-Small-LF-V3) can introduce a noticeable performance drop, underscoring the inherent trade-off between compression ratio and accuracy. Second, our knowledge distillation approach leverages four distinct losses—embedding-layer, hidden-state, output-layer, and attention-based distillation. By aligning intermediate representations with the teacher model, the student model (KR-ELECTRA-Small-KD) preserves a large portion of the teacher’s performance and even surpasses it on the NSMC task. This result suggests that cross-layer representation learning is beneficial for languages like Korean, which exhibit complex morphological structures. Nevertheless, the knowledge distillation efficacy depends heavily on the teacher model’s capacity, leaving room for further improvement should a larger teacher become available. Third, while our experiments covered six widely used Korean NLP datasets, additional tasks—such as question generation, dialog modeling, or domain-specific classification—may further validate the model’s robustness. Moreover, real-world latency tests on various mobile or embedded hardware would offer practical insights into the deployment feasibility of each factorized variant. Overall, the proposed approach demonstrates that systematically combining knowledge distillation with selective low-rank factorization can yield a family of compact Korean language models that balance inference efficiency and task performance.

### 3.5. Limitations

Despite the promising results achieved by our proposed models, it is important to acknowledge some limitations of this study. First, the scope of our hyperparameter search was constrained by computational limitations. Consequently, it is plausible that a more exhaustive exploration of the hyperparameter space could lead to the discovery of even more performant model configurations. Second, our evaluation was confined to six specific Korean NLP tasks. Although these tasks encompass a variety of language understanding capabilities, future research should broaden the scope of evaluation to include a more diverse set of tasks. This would enable a more comprehensive assessment of the models’ generalizability and overall performance. Third, the efficacy of our student models is intrinsically linked to the quality of the teacher model. Thus, a more capable teacher model could potentially yield even more effective student models through the knowledge distillation process. Finally, while our models exhibit substantial reductions in size, it is crucial to acknowledge the trade-off between model size and performance, especially when considering specific application. Future research should delve into determining the optimal balance between these two factors for a variety of resource-constrained scenarios.

## 4. Conclusions

We presented lightweight Korean pre-trained language models developed using knowledge distillation and low-rank factorization. Our approach effectively creates compact models suitable for resource-constrained environments. Experiments demonstrated that our smallest model (KR-ELECTRA-Small-LF-V3, 18 MB) retains over 93.9% of the 110 M parameter teacher’s performance on average across six Korean NLP tasks, representing a 24× size reduction. Furthermore, the knowledge-distilled model (KR-ELECTRA-Small-KD) slightly outperformed the teacher on the NSMC task. These results validate the potential of combining knowledge distillation and low-rank factorization for efficient Korean NLP. Future work could explore broader task evaluations and refined compression techniques.

## Figures and Tables

**Table 1:** Number of parameters in ELECTRA-base model with base model size.

Structure of ELECTRA	Number of Parameters	Percentage of Total
Feed-Forward Network	56,623,104	50%
Embedding Layer	24,576,000	22%

**Table 2:** Number of parameters in lightweight ELECTRA variants.

Structure of Lightweight ELECTRA	Number of Parameters	Value of r
Lightweight Feed-Forward Network	14,155,776	$\frac{m}{4}$
Lightweight Embedding Layer	6,144,000	$\frac{m}{4}$

**Table 3:** List of hyperparameters in pre-training.

Hyperparameter	Model Size		
	Tiny	Small	Base
Number of Layers	12	12	12
Hidden Size	128	256	768
FFN Inner Hidden Size	512	1024	3072
Attention Heads	2	4	12
Attention Head Size	64	64	64
Embedding Size	64	128	768
Parameters	4 M	14 M	110 M
Model Size	17 MB	53 MB	432 MB

**Table 4:** List of fine-tuning hyperparameters for downstream tasks.

Task	Learning Rate	Batch Size	Epochs	Early Stopping
NSMC	3 × 10^−5^	32	3	No
KoHate	3 × 10^−5^	32	3	No
KorNLI	3 × 10^−5^	32	3	No
KorSTS	3 × 10^−5^	32	3	No
NER	3 × 10^−5^	16	5	Yes
KorQuAD	3 × 10^−5^	8	2	No

**Table 5:** Example from NSMC dataset.

Sentence and Meaning		Label
Koreans	아 더빙.. 진짜 짜증나네요 목소리	Negative
Meaning	Dubbing.. It’s really annoying voice	
Koreans	액션이 없는데도 재미 있는 몇 안되는 영화	Positive
Meaning	One of the few movies that is fun without action	

**Table 6:** Example from KorNLI dataset. P: Premise; H: Hypothesis.

Sentence and Meaning		Label
Koreans	P: 저는, 그냥 알아내려고 거기 있었어요. H: 이해하려고 노력하고 있었어요.	Entailment
Meaning	P: I was there just to find out. H: I was trying to understand.	
Koreans	P: 저는, 그냥 알아내려고 거기 있었어요. H: 나는 처음부터 그것을 잘 이해했다.	Contradiction
Meaning	P: I was there just to find out. H: I understood it well from the beginning.	
Koreans	P: 저는, 그냥 알아내려고 거기 있었어요. H: 나는 돈이 어디로 갔는지 이해하려고 했어요.	Neutral
Meaning	P: I was there just to find out. H: I was trying to understand where the money went.	

**Table 7:** Performance comparison of KR-ELECTRA-Small-KD with different β values and the teacher model (KR-ELECTRA-Base) on six Korean NLP tasks. Bold indicates the best performance among the student models. Red indicates performance higher than the teacher model.

Model	Model Size (MB)	Hyperparameter β	Data						Avg
			NSMC (ACC)	Naver NER (F1)	KorNLI (ACC)	KorSTS (Spearman)	KorQuaD (EM/F1)	Korean HateSpeech (F1)	
KR-ELECTRA-Base *	432	1.0	89.324	87.896	80.878	81.722	59.369/ 88.993	66.273	79.208
KR-ELECTRA-Small	53	1.0	88.798	85.409	77.485	76.809	57.256/ 86.544	61.598	76.271
KR-ELECTRA-Small-KD	53	0.3	**89.262**	**85.432**	**77.764**	**77.367**	57.066/ **86.716**	64.236	**76.835**
	53	0.5	**89.720**	**85.873**	**78.223**	76.076	**57.637/** **87.143**	**65.302**	**77.139**
	53	0.7	**89.428**	**85.603**	77.365	75.424	**57.516/** **86.865**	**64.301**	**76.643**

* Teacher model.

**Table 8:** Performance comparison of KR-ELECTRA-Small-LF-V1 with different β values. Boldface indicates performance exceeding that of the KR-ELECTRA-Small-LF-V1 model, while red indicates the highest performance among the student models, excluding the teacher model.

Model	Model Size (MB)	Hyperparameter β	Data						Avg
			NSMC (ACC)	Naver NER (F1)	KorNLI (ACC)	KorSTS (Spearman)	KorQuaD (EM/F1)	Korean HateSpeech (F1)	
KR-ELECTRA-Base *	432	1.0	89.324	87.896	80.878	81.722	59.369/ 88.993	66.273	79.208
KR-ELECTRA-Small-LF-V1	35	1.0	88.062	82.965	74.730	73.460	**53.983/** **83.125**	61.165	73.927
KR-ELECTRA-Small-LF-V1-KD	35	0.3	**88.614**	**83.769**	**75.089**	**74.463**	51.974/ 81.110	60.783	73.686
	35	0.5	**89.012**	**84.983**	**75.828**	**73.889**	53.134/ 82.477	**63.389**	**74.673**
	35	0.7	**88.726**	**83.99**	**74.750**	**73.728**	53.186/ 82.256	60.877	**73.930**

* Teacher model.

**Table 9:** Performance comparison of KR-ELECTRA-Small-LF-V2 with different β values. Boldface indicates performance exceeding that of the KR-ELECTRA-Small-LF-V1 model, while red indicates the highest performance among the student models, excluding the teacher model.

Model	Model Size (MB)	Hyperparameter β	Data						Avg
			NSMC (ACC)	Naver NER (F1)	KorNLI (ACC)	KorSTS (Spearman)	KorQuaD (EM/F1)	Korean HateSpeech (F1)	
KR-ELECTRA-Base *	432	1.0	89.324	87.896	80.878	81.722	59.369/ 88.993	66.273	79.208
KR-ELECTRA-Small-LF-V2	30	1.0	87.898	81.517	74.151	72.091	51.437/ 80.142	60.734	72.567
KR-ELECTRA-Small-LF-V2-KD	30	0.3	**88.248**	**84.152**	**75.069**	**74.845**	**55.143/** **84.685**	**62.048**	**74.884**
	30	0.5	**88.884**	**84.922**	**75.608**	**73.899**	**56.096/** **85.810**	**63.612**	**75.547**
	30	0.7	**88.346**	**84.614**	**76.147**	**73.514**	**55.438/** **84.713**	**64.132**	**75.272**

* Teacher model.

**Table 10:** Performance comparison of KR-ELECTRA-Small-LF-V3 with different β values. Boldface indicates performance exceeding that of the KR-ELECTRA-Small-LF-V1 model, while red indicates the highest performance among the student models, excluding the teacher model.

Model	Model Size (MB)	Hyperparameter β	Data						Avg
			NSMC (ACC)	Naver NER (F1)	KorNLI (ACC)	KorSTS (Spearman)	KorQuaD (EM/F1)	Korean HateSpeech (F1)	
KR-ELECTRA-Base *	432	1.0	89.324	87.896	80.878	81.722	59.369/ 88.993	66.273	79.208
KR-ELECTRA-Tiny	17	1.0	87.454	78.878	72.355	71.019	51.627/ 80.791	59.120	71.606
KR-ELECTRA-Small-LF-V3	18	1.0	87.506	81.906	73.912	71.302	51.783/ 80.626	61.862	72.700
KR-ELECTRA-Small-LF-V3-KD	18	0.3	**88.378**	**83.439**	**75.369**	**72.930**	**54.624/** **84.094**	**62.129**	**74.423**
	18	0.5	**88.138**	**83.537**	**74.011**	**74.220**	**54.121/** **83.528**	**63.571**	**74.447**
	18	0.7	**88.374**	**84.237**	73.333	**73.004**	**54.104/** **83.443**	61.362	**73.980**

* Teacher model.

**Table 11:** Inference time comparison on CPU.

Model	Inference Time (ms/Sentence)
KR-ELECTRA-Base	12.5
KR-ELECTRA-Small-KD	3.2
KR-ELECTRA-Small-LF-V1	2.1
KR-ELECTRA-Small-LF-V2	1.8
KR-ELECTRA-Small-LF-V3	1.1

**Table 12:** Ablation study results, comparing performance with and without low-rank factorization (LF) on FFN.

Model Configuration	NSME (ACC)	NER (F1)	KorNLI (ACC)	KorSTS (Spearman)	KorQuAD (EM/F1)	Korean HateSpeech (F1)	Avg	Model Size
Without LF (KD only)	89.720	85.873	78.223	76.076	57.637/87.143	65.302	77.139	52 MB
With LF (KD + LF-FFN)	89.012	84.983	75.828	73.889	53.134/82.477	63.389	74.673	35 MB

## Data Availability

The data presented in this study are openly available in https://github.com/e9t/nsmc (accessed on 31 March 2025).
